# Emerging tools for the early detection of prostate cancer

**DOI:** 10.1002/bco2.70081

**Published:** 2025-09-18

**Authors:** Muhammad Haider, Jeffrey J. Leow, Tobias Nordström, Ashkan Mortezavi, Peter Albers, Rakesh Heer, Prabhakar Rajan

**Affiliations:** ^1^ Centre for Cancer Cell and Molecular Biology Barts Cancer Institute, Queen Mary University of London UK; ^2^ Department of Urology Barts Health NHS Trust London UK; ^3^ Department of Urology University College London Hospitals NHS Foundation Trust London UK; ^4^ Department of Urology Tan Tock Seng Hospital Singapore; ^5^ Lee Kong Chian School of Medicine Nanyang Technological University Singapore; ^6^ Department of Clinical Sciences, Danderyds Sjukhus Stockholm Sweden; ^7^ Department of Medical Epidemiology and Biostatistics Karolinska Institutet Stockholm Sweden; ^8^ Department of Urology University Hospital Zürich Zürich Switzerland; ^9^ Department of Urology University Hospital Düsseldorf Düsseldorf Germany; ^10^ Department of Personalized Early Detection of Prostate Cancer German Cancer Research Center Heidelberg Germany; ^11^ Division of Surgery, Imperial College Imperial Prostate London UK; ^12^ Department of Urology Imperial College Healthcare NHS Trust London UK

**Keywords:** 4 K score, AI, early diagnosis, ExoDx prostate, MRI, MyProstateScore, polygenic risk score, prostate cancer, prostate health index, PRS, PSA, screening, SelectMDx, Stockholm 3, ultrasound

## Abstract

**Introduction:**

Prostate cancer (PCa) is the second most common cancer in men globally, with a rising incidence. Early detection through population‐based screening by Prostate Specific Antigen (PSA) testing improves survival outcomes, at the expense of overdiagnosis and overtreatment of clinically insignificant disease. Here, we explore emerging tools for more effective PCa early detection and evaluate their potential roles for PCa screening.

**Materials and Methods:**

Key articles on emerging adjuncts and alternatives to PSA for PCa early detection were identified.

**Results:**

Multiparametric MRI (mpMRI) remains the gold standard modality for identifying clinically significant PCa and has been evaluated for screening. Newer imaging strategies incorporating biparametric MRI (bpMRI) or multiparametric ultrasound (mpUS) potentially offer similar accuracy to mpMRI. Saliva‐derived polygenic risk scores (PRS) hold potential as a non‐invasive screening tool to identify at‐risk patient groups. Blood‐based biomarker tests can improve risk stratification, reducing unnecessary biopsies while maintaining detection of clinically significant cancers compared to PSA alone. Urine‐based biomarker tests have been examined for the early detection and risk stratification of clinically significant disease as adjuncts to PSA testing.

**Conclusion:**

PSA is commonly used to detect early PCa, but its lack of specificity and associated overdiagnosis risk has led to controversy over its use for population‐based screening. Imaging modalities such as mpMRI have reduced detection of clinically insignificant PCa, and emerging cost‐effective alternatives, such as bpMRI and mpUS, show promise. Molecular biomarkers and PRS for risk stratification may help target imaging‐based early detection more effectively to at‐risk populations. Prospective randomised clinical trials are urgently needed to evaluate the performance of different modalities for population‐wide screening. Future developments may involve technologies such as artificial intelligence and diagnostic tests that incorporate circulating tumour markers.

**Abbreviations**
PSA: Prostate Specific Antigen
csPCa: Clinically Significant Prostate Cancer
mpMRI: Multiparametric Magnetic Resonance Imaging
bpMRI: Biparametric Magnetic Resonance Imaging
mpUS: Multiparametric Ultrasound
Stockholm3: Stockholm3
DRE: Digital Rectal Exam
AUC: Area Under the Curve
PHI: Prostate Health Index
NICE: National Institute for Health and Care Excellence
PI‐RADS: Prostate Imaging Reporting and Data System
GG: Gleason Grade
PCPTRC: Prostate Cancer Prevention Trial Risk Calculator
ESRPC‐RC: European Randomised Study of Screening for Prostate Cancer Risk Calculator
PCA3: Prostate Cancer Antigen 3
T2:ERG: TMPRSS2:ERG Gene Fusion
MPS: MyProstateScore
CEUS: Contrast‐Enhanced Ultrasound
CUDI: Contrast‐Ultrasound‐Dispersion Imaging
MUSIC‐Screen: A Phase 3, Multicentre, International, Non‐inferiority, Randomised Clinical Trial Comparing Screening for Prostate Cancer Using High Resolution Micro‐ultrasound Versus Multiparametric Magnetic Resonance Imaging (MUSIC‐Screen)
OPTIMUM: Optimisation of Prostate Biopsy ‐ Micro‐Ultrasound versus MRI: a 3‐arm randomised controlled trial evaluating the role of 29 MHz micro‐ultrasound in guiding prostate biopsy in men with clinical suspicion of prostate cancer
ISUP: International Society of Urological Pathology
miRNA: MicroRNA
PRS: Polygenic Risk Scores
PLCO: Prostate, Lung, Colorectal and Ovarian
ERSPC: European Randomised Study of Screening for Prostate Cancer
CAP: Cluster Randomised Trial of PSA testing for Prostate Cancer
PROMIS: PROstate MRI Imaging Study
PRECISION: Prostate Evaluation for Clinically Important Disease: Sampling Using Image‐guidance or Not?
MRI‐FIRST: Use of prostate systematic and targeted biopsy on the basis of multiparametric MRI in biopsy‐naive patients
IP7‐PACIFIC: Imperial Prostate 7 ‐ Prostate Assessment Using Comparative Interventions ‐ Fast MRI and Image‐fusion for Cancer
VISIONING: Evaluation of a MRI‐based Prostate Cancer Screening Program
PRAISE‐U: Prostate Cancer Awareness and Initiative for Screening in the European Union
MVP: Prostate MRI versus PSA Screening for Prostate Cancer Detection
IP‐1 PROSTAGRAM: Population‐Based Prostate Cancer Screening With Magnetic Resonance Imaging or Ultrasonography
PROBASE: German Prostate Cancer Early Detection Study Based on a Baseline PSA Value in Young Men
CADMUS: Cancer Diagnosis by Multiparametric Ultrasound of the Prostate
BARCODE1: Biomarker for Risk of Prostate Cancer: 1st Study
PROFILE: Germline Genetic Profiling: Correlation with Targeted Prostate Cancer Screening and Treatment
BPH: Benign Prostatic Hyperplasia
EU: European Union
4Kscore: Four Kallikrein Score

## 1 INTRODUCTION

Prostate cancer (PCa) is the second most common cancer in men globally.^1^ In 2020, 1.4 million men were diagnosed with PCa worldwide, and this number is expected to rise to 2.9 million by 2040, driven by factors such as increasing life expectancy and population growth.^2^ PCa is the fifth leading cause of cancer‐related deaths globally,^1^ with most deaths occurring due to late diagnosis, as early‐stage disease has excellent cure rates with radical treatment options.^3^

Serum prostate‐specific antigen (PSA) has been the most widely used first‐line test for PCa detection. PSA (encoded by *KLK3*) is a serine protease glycoprotein that liquefies the seminal coagulum.^4^ However, while PSA is organ‐specific, it is not cancer‐specific and can be elevated in benign conditions like BPH and prostatitis.^5^ Only one‐third of men with elevated PSA will have a diagnosis of cancer on initial assessment.^6^ Moreover, of those diagnosed with PCa, the majority (69.4%) are diagnosed with localised cancer,^7^ of which approximately half are diagnosed with clinically insignificant PCa (Gleason score ≤6).^8^ For men diagnosed with Gleason ≤6 tumours and classified as low risk (PSA < 10 ng/ml and stage ≤ T2a), the mortality from PCa over a 10‐ to 15‐year period, even without any intervention, is less than 3%,^9^ and therefore the risks of treatment often outweigh the benefits.

Large PSA‐based screening trials have shown mixed results for the reduction of PCa mortality.^10^ This may be explained by methodological issues in trials such as the PLCO Cancer Screening trial, which failed to show any benefit of organised annual PSA testing.^11^ However, the control group had significant contamination, with men undergoing PSA testing outside the trial, averaging 2.7 tests in the control group compared to 5 in the PSA testing group.^12^ The ERSPC trial showed a 20% reduction in mortality after a 16‐year follow‐up, equating to 18 diagnoses needed to prevent one death. There were also a large number of unnecessary biopsies, with only 24% of biopsies leading to a PCa diagnosis.^13^ A large UK study, the CAP trial, showed that one‐off PSA screening led to an increase in the number of diagnoses compared to the control group (4.3% vs. 3.6%, respectively). However, this did not lead to a statistically significant difference in PCa mortality rates (rate ratio 0.96, 95% CI 0.85 to 1.08) after a median follow‐up of 10 years.^14^ Due to unclear evidence that PCa screening benefits outweigh harms, the USPSTF advises against population‐wide screening, recommending a shared decision‐making process for men aged 55–69 with their clinician after weighing risks and benefits.^15^

To address the limitations of PSA‐based testing, new imaging, genetic and molecular biomarker tests have been developed as adjuncts or alternatives. The aim is to create a cost‐effective test with high sensitivity and specificity for csPCa while minimising overdiagnosis of clinically insignificant tumours. Here, we examine and evaluate the evidence for these emerging tools and their suitability for early PCa detection.

## 2 EMERGING ADJUNCTS AND ALTERNATIVES TO PSA FOR PCA EARLY DETECTION

A summary of the alternative or adjunct tests to PSA for the early detection of PCa is provided in Table 1.

**TABLE 1:** Summary of the alternative or adjunct tests to PSA for the early detection of prostate cancer (PCa).

Imaging tests				
Name	Information		When to use	Referenced studies
**Magnetic Resonance Imaging (MRI)**	Multiparametric MRI (mpMRI) includes the following phases: T2‐weighted imaging (T2WI), Diffusion‐weighted imaging (DWI), Dynamic contrast‐enhanced imaging (DCE). (DCE phase is not used in bpMRI) Likert and Prostate Imaging–Reporting and Data System (PI‐RADS) scoring systems are used to assess and standardise mpMRI findings.		Can be as used pre‐biopsy for detecting suspicious lesions and to aid targeted biopsy.	10–20
**Ultrasound**	Multiparametric ultrasound (mpUSS) includes contrast‐enhanced ultrasound (CEUS), micro‐Doppler and real‐time elastography. There is currently no universally accepted standardised scoring system for multiparametric ultrasound (mpUSS) in prostate cancer detection. ‐			21–27
**Serum tests**				
**Name**	**Biomarkers**	**Clinical Data**	**When to use**	**Referenced studies**
**Stockholm3** **(STHLM3)**	5 Protein markers (human glandular kallikrein 2, microseminoprotein beta, microphage inhibitory cytokine‐1, total PSA and free PSA) & 101 genetic Single Nucleotide Polymorphisms (SNPs)	Age, family history and previous prostate biopsy	45–74 years old with no previous prostate cancer diagnosis and PSA ≥ 1.5	28–33
**4Kscore®**	Four prostate specific kallikreins (Total PSA, Free PSA, Intact PSA and human kallikrein 2)	Age, DRE, previous negative biopsy	≥ 45 years old with abnormal PSA/DRE prior to 1st biopsy or after a negative biopsy	34–38
**Prostate Health Index (PHI)**	Three kallikrein assays (PSA, free PSA and [−2]proPSA)	‐	≥ 50 years old with PSA 2–10 and negative DRE	40–46
**Urine tests**				
**Name**	**Biomarkers**	**Clinical Data**	**When to use**	**Referenced studies**
**ExoDx**	Exosomal RNA expression of 3 genes – ERG, PCA3 & SPDEF	‐	men ≥50 years old with PSA levels 2–10 for initial/repeat biopsy	47–49
**My ProstateScore** **(MPS)**	Urinary PCA3 & TMPRSS2:ERG (post DRE) and serum PSA	‐	Estimates risk of Gleason Grade ≥ 2 tumours on biopsy	50–51
**SelectMDx**	mRNA expression of DLX1 (Distal‐Less Homeobox 1) and HOXC6 (Homeobox protein Hox‐C6) (Post‐dre)	Age, DRE, PSA, prostate volume	Estimates risk of any prostate cancer & Gleason score ≥ 7 on biopsy	52–57

### 2.1 Imaging

#### 2.1.1 Multi‐parametric (mp) magnetic resonance imaging (MRI)

Multiparametric MRI (mpMRI) of the prostate has been the gold standard tool for identification of clinically significant prostate cancer (csPCa).^16^ It consists of multiple sequences including anatomic (T1 and T2 weighted) and functional sequences including dynamic contrast‐enhanced (DCE) and diffusion‐weighted imaging (DWI).^17^ Current European Association of Urology (EAU) guidelines strongly recommend its use prior to biopsy and the use of the Prostate Imaging Reporting & Data System (PI‐RADS) scoring system (Table 2) to assess mpMRI features to determine if a biopsy is warranted.^18^ An alternative recommended by the UK National Institute for Health and Care Excellence (NICE)^19^ is the Likert scoring system (Table 2).

**TABLE 2:** Summary of PI‐RADS v2.1 and Likert scoring systems.

		PI‐RADS v2.1 (1)‐ each lesion is scored from 1 to 5 on diffusion weighted and T2‐weighted sequences and presence of dynamic contrast enhancement.					Likert (2)‐ the Likert scoring system does not use fixed criteria. Instead, it is based on an overall impression		
Score	Meaning	T2‐weighted Peripheral Zone	T2‐weighted Transition Zone	Diffusion weighted imaging (Peripheral or Transitional zone)	Dynamic Contrast Enhancement (DCE)	Cancer detection rate for any PCa & (csPC)	Likert Score	Meaning	Cancer detection rate for any PCa & (csPC)^3^
**1**	Very low (clinically significant cancer is highly unlikely to be present)	Uniform high signal intensity (normal)	Normal appearing transition zone (rare) or a round, completely encapsulated nodule (“typical nodule” of benign prostatic hyperplasia)	No abnormality on ADC or high b‐value DWI	(−) negative: no early or contemporaneous enhancement, or diffuse multifocal enhancement not corresponding to a focal finding on T2W and/or DWI, or focal enhancement corresponding to a lesion demonstrating features of benign prostatic hyperplasia on T2W (including features of extruded benign prostatic hyperplasia nodule in the peripheral zone) (+) positive: focal, and earlier than or contemporaneous with enhancement of adjacent normal prostatic tissues, and corresponds to suspicious finding on T2 and/or DWI For peripheral zone lesions with DWI score of 3, the presence of dynamic contrast enhancement is used to upgrade the overall PI‐RADS assessment category to 4.	3% (2%)	1	Highly unlikely to be clinically significant cancer	12% (4%)
**2**	Low (clinically significant cancer is unlikely to be present)	Linear or wedge‐shaped hypointensity or diffuse mild hypointensity, usually indistinct margin	A mostly encapsulated nodule or a homogeneous circumscribed nodule without encapsulation (“atypical nodule”), or a homogeneous mildly hypointense area between nodules	Linear/wedge shaped, hypointensity on ADC and/or hyperintensity on high b‐value DWI		9% (4%)	2	Unlikely to be clinically significant cancer	13% (4%)
**3**	Intermediate (the presence of clinically significant cancer is equivocal)	Heterogeneous signal intensity or non‐circumscribed, rounded, moderate hypointensity; includes others that do not qualify as 2, 4, or 5	lenticular or non‐circumscribed, homogeneous, moderately hypointense, and <1.5 cm in greatest dimension	Focal (discrete and different from background), mild/moderate hypointensity on ADC and/or mild/moderate hyperintensity on high b‐value DWI; may be markedly hypointense on ADC or markedly hyperintense on high b‐value DWI, but not both		34% (20%)	3	Equivocal, moderate likelihood of clinically significant cancer	22% (12%)
**4**	High (clinically significant cancer is likely to be present)	Circumscribed, homogeneous, moderate hypointensity and <1.5 cm in greatest dimension	Lenticular or non‐circumscribed, homogeneous, moderately hypointense and <1.5 cm in greatest dimension	Focal, marked hypointensity on ADC and marked hyperintensity on high b‐value DWI; <1.5 cm in greatest dimension		70% (52%)	4	Likely to be clinically significant cancer	50% (33%)
**5**	Very high (clinically significant cancer is highly likely to be present)	Same as 4 but ≥1.5 cm in greatest dimension or definite extraprostatic extension/invasive behaviour	Same as 4, but ≥1.5 cm in greatest dimension or definite extraprostatic extension/invasive behaviour	Same as 4 but ≥1.5 cm in greatest dimension or definite extraprostatic extension/invasive behaviour		97% (89%)	5	Highly likely to be clinically significant cancer	59% (48%)
**X**	Component of exam technically inadequate or not performed								

References

1.Czarniecki M, Le L, Knipe H, et al. Prostate Imaging‐Reporting and Data System (PI‐RADS). Reference article, Radiopaedia.org. 2024; Available at: https://radiopaedia.org/articles/27968. Accessed Accessed on 11 Dec, 2024.

2.Rosenkrantz AB, Lim RP, Haghighi M, Somberg MB, Babb JS, Taneja SS. Comparison of interreader reproducibility of the prostate imaging reporting and data system and likert scales for evaluation of multiparametric prostate MRI. Am J Roentgenol 2013;201^4^:W612–W618.

3.Shin T, Smyth TB, Ukimura O, Ahmadi N, de Castro Abreu AL, Ohe C, et al. Diagnostic accuracy of a five‐point Likert scoring system for magnetic resonance imaging (MRI) evaluated according to results of MRI/ultrasonography image‐fusion targeted biopsy of the prostate. BJU Int 2018;121^1^:77–83.

The findings of key prostate diagnostic trials have cemented the place of mpMRI imaging prior to prostate biopsy. The PROMIS trial showed that mpMRI pre‐biopsy could reduce the need for biopsy in 27% of patients compared to PSA alone and reduce the diagnosis of clinically‐insignificant PCa.^20^ The PRECISION study demonstrated that mpMRI‐guided biopsy was non‐inferior to the standard TRUS‐guided biopsy pathway, leading to a 12% increase (p = 0.005) in the detection of csPCa and reducing the number of biopsies by 28%.^21^ Following on from this, the MRI‐FIRST study demonstrated that the combination of systematic and targeted biopsies achieved a greater detection rate of csPCa compared to using either technique alone.^22^

#### 2.1.2 Biparametric (bp) MRI

Biparametric (bpMRI) eliminates the use of gadolinium contrast, thereby removing the DCE sequences, which has time and cost implications. The Prostate Imaging Using MRI +/​‐ Contrast Enhancement (PRIME) large (N = 490) multi‐centre trial comparing bpMRI to mpMRI for detecting csPCa and was presented at EAU24.^23^ The proportion of patients requiring biopsy was similar for both bpMRI 273/490 (56%) and mpMRI 279/490 (57%). bpMRI was non‐inferior to mpMRI for detecting csPCa (Gleason ≥3 + 4), leading to only a 0.4% lower detection rate (143/490, 29.2%) for mpMRI versus 141/490, 28.8% for bpMRI) (p = 0.5). Additionally, there was no significant difference in the sensitivity (99.3% and 97.9% for mpMRI and bpMRI, respectively) and specificity (59.9% and 61.1% for mpMRI and bpMRI, respectively) between the two modalities. To ensure that only high‐quality MRIs were included in the trial, only centres with an MRI quality score of 4 or 5, according to the Prostate Imaging Quality (PI‐QUAL) standardised scoring system, were selected.^24^ In Phase I, only 23 out of 71 scanners (32.4%) initially achieved a PI‐QUAL score of 5. After implementing targeted recommendations to improve MRI protocols, such as adjustments to image resolution, contrast timing and sequence parameters, 62 out of 64 scanners (96.9%) achieved a PI‐QUAL score of 5 in Phase II. These data demonstrate that systematic evaluation and protocol optimisation can significantly enhance prostate MRI quality. Future trials should prioritise high‐quality MRI imaging to ensure reproducibility. The IP7‐PACIFIC trial is currently underway as a definitive clinical utility study and health economic evaluation of bpMRI and mpMRI, as well as visual registration versus image‐guided fusion biopsy.^25^

#### 2.1.3 Ultra‐short bpMRI

In the context of population‐based organised screening availability, the costs of MRI may become a limitation. Therefore, shorter protocols would enhance accessibility and reduce the costs of imaging. A Dutch study compared prospectively mpMRI with triplanar noncontrast MRI (standard bpMRI) and a fast monoplanar noncontrast MRI (fast bp‐MRI).^26^ The study found that all protocols had a sensitivity of 95% for detecting high‐grade PCa. However, fast bp‐MRI slightly reduced specificity (65% vs. 69%) and led to a marginal increase in unnecessary biopsies and low‐grade PCa detection. The sequence times per protocol were 16 min for mpMRI, 13 min for bpMRI and 8 min for fast bpMRI.

#### 2.1.4 MRI for population‐based PCa screening

Although current EAU guidelines advise against using MRI for PCa screening (18), emerging evidence suggests that MRI‐based imaging may be an alternative to PSA‐based screening. Furthermore, the EAU is collaborating with the EU on the EAU project known as PRAISE‐U.^27^ The project aims to reduce morbidity and mortality in PCa by developing improved screening strategies, including the use of the combination of PSA and MRI for PCa screening.

The Göteborg 2 study (N = 17 980) showed that the use of MRI for screening could reduce overdiagnosis of insignificant PCa by half.^28^ Men with raised PSA (≥3 ng/ml) were assigned to one of two groups. The reference group underwent systematic and targeted biopsies, while the experimental group underwent MRI‐targeted biopsies only. In the experimental group, the risk of overdiagnosis of clinically insignificant PCa was reduced by half compared to the reference group (0.6% versus 1.2%; RR 0.46; 95% CI, 0.33–0.64; P < 0.001), but this was at the expense of missing some csPCa (0.9% versus 1.1%; RR 0.81; 95% CI, 0.60–1.1). However, in the reference group, all csPCa (10 patients) detected by systematic biopsy only were classified as intermediate risk (based on the modified National Comprehensive Cancer Network classification) and were managed with active surveillance. An important limitation of this study is that the NPV was not evaluated in the experimental group, as men with PIRADS 1–2 did not receive a biopsy and there was no extended follow‐up for these men.

More recently, a large systematic review and meta‐analysis, which included data from over 80,000 men across 12 studies, found that integrating MRI into PCa screening pathways reduces the number of unnecessary biopsies and the overdiagnosis of insignificant PCa, while maintaining the detection rates of csPCa compared to PSA‐only screening.^29^

The MVP trial looked at using bpMRI‐only screening compared to PSA in Canada.^30^ BpMRI had superior performance with a 63% cancer detection rate (15/24) vs. 29% (8/23) (p = 0.019) for PSA only. bpMRI also detected more csPCa cases than PSA alone (73% vs. 50%), with a relative risk of 2.77 (95% CI 0.89 to 8.59, p = 0.07). More recently, the ReIMAGINE trial looked at the role of a screening bpMRI (consisting of a T2‐weighted and diffusion‐weighted sequence) in screening. A total of 303 men underwent MRI, and 48 (16%) men had a positive MRI, and of those, 25(52%) had csPCa, and only 2(4%) had clinically insignificant cancer. Interestingly, they found that 2/3 of men with a positive MRI and 60% of men who had csPCa had a PSA <3 ng/ml.^31^

A prospective cohort study (IP‐1 PROSTAGRAM study, N = 408) compared PSA, bpMRI and ultrasound (US), consisting of b‐mode and shear wave elastography, for PCa screening.^32^ The number of positive results was similar when a PSA cutoff of ≥3 was compared to bpMRI and US cutoff scores of 4–5. Specifically, 40 patients (9.9%; 95% CI, 7.3%–13.2%) had elevated PSA, 43 (10.6%; 95% CI, 7.9%–14.0%) had abnormal bpMRI findings and 52 (12.8%; 95% CI, 9.9%–16.5%) had abnormal US findings. Using these cutoff scores, bpMRI detected the most csPCa (11), followed by PSA (7) and US (4).

The Swiss VISIONING trial investigated the use of bpMRI as primary opportunistic screening for PCa without using a PSA cut‐off.^33^ Conducted on biopsy‐naïve men over 45, the study found that bpMRI could detect csPCa in cases that would otherwise have been missed by PSA and DRE (Digital Rectal Examination) alone. With a total of 229 participants, 21 were found to have csPCa, yielding a detection rate of approximately 1 in every 11 bpMRI scans. Of note, the median PSA value in the cohort was 1.26 ng/ml. A protocol adjustment deferring biopsies for PI‐RADS 3 lesions unless they showed persistence or upgrading further reduced the number of unnecessary biopsies.

Whilst MRI‐based screening has been increasingly explored for PCa detection, this strategy has limitations including interobserver variability and cost.^34^ Efforts to address these limitations include a UK‐based study using luminal index MRI (LI‐MRI) for community screening. LI‐MRI offers shorter scan times (5 minutes) and lower costs.^35^ Additionally, the PRAISE‐U project conducted a systematic review examining the cost‐effectiveness of PCa screening in Europe.^36^ Screening studies involving initially healthy populations found a median incremental cost‐effectiveness ratio (ICER) of €56487 per quality‐adjusted life year (QALY), with a range from €5872 to €372948 per QALY. Furthermore, the review highlighted that risk‐based screening strategies incorporating MRI were more cost‐effective compared to no screening.

MRI screening may also not be the optimal modality for detecting PCa in younger men, as demonstrated by the PROBASE trial.^37^ Although this trial did not solely evaluate imaging‐based screening but rather risk‐adapted PCa screening based on baseline PSA, it underscored the limitations of mpMRI for PCa detection in younger men. Interpreting MRIs in younger patients is more complex due to age‐related changes, making it more difficult to exclude cancer. In the group starting screening at age 45, a high proportion of PI‐RADS 3 lesions were identified (43% and 48% for local and reference reporting, respectively) and among the 49 patients classified as PI‐RADS 3 on local reading, 20% had csPCa. However, in the Swiss Visioning trial, a significant number of these men were downgraded to a PI‐RADS 1 or 2 on follow‐up MRIs.^33^

#### 2.1.5 Ultrasound

Emerging advances in ultrasound (US) technologies are improving the diagnostic accuracy of PCa detection.^38^ Compared to MRI, US offers several advantages, including lower cost, real‐time imaging, convenience and suitability for patients with contraindications to MRI.^39^ Additionally, US can guide biopsies and local treatments such as high‐intensity focused ultrasound (HIFU).^39^, ^40^ Limitations of US include operator dependence and lack of readily available images for review of equivocal cases.

Advancements in the US include contrast‐enhanced ultrasound (CEUS), micro‐Doppler and real‐time elastography. When used in combination, this approach is termed “multiparametric ultrasound” (mpUS) and has yielded promising results in trials such as the CADMUS study.^41^ This large multi‐centre prospective study, patients with either raised PSA or abnormal DRE underwent both mpUS and mpMRI and a subsequent biopsy if either scan was positive. One hundred and thirteen patients were diagnosed with PCa, out of which 83 had csPCa, MpUS detected 4.3% less csPCa compared to mpMRI (66 vs 77 patients detected, respectively) and would have led to an additional 11.1% of patients undergoing biopsy.

Another prospective single centre trial compared the use of mpMRI and targeted biopsy (TBx), contrast‐ultrasound‐dispersion imaging (CUDI)‐TBx and systematic biopsies.^42^ There was no statistically significant difference between the two imaging modalities for the detection of csPCa, 41/142 (29%) and 40/142 (28%) in mpMRI and CUDI groups, respectively. However, the trial was stopped due to both pathways performing worse than the systematic biopsies, which detected significantly more csPCa: 56/142 (39%) (p = <0.05).

One meta‐analysis looked at the use of micro‐US (300% higher resolution than traditional US systems) versus mpMRI and found that both modalities were comparable with no statistically significant difference in sensitivity or specificity between the two modalities.^43^ The use of micro‐US has also been compared to systematic biopsies in a meta‐analysis, which showed that, compared to systematic biopsies, micro‐US identified more cases of csPCa, with 196 versus 169 cases respectively (Detection Ratio [DR]: 1.18, 95% CI 0.83–1.68, I^2^ = 69%). Additionally, micro‐US guided biopsies detected fewer clinically insignificant cancers, with 62 cases compared to 115 cases for systematic biopsies (DR: 0.55, 95% CI 0.41–0.73, I^2^ = 0%).^44^ These findings were further corroborated in the first multicentre prospective trial to compare micro‐US and mpMRI targeted biopsies.^45^ Micro‐US guided biopsies were shown to be non‐inferior for the detection of csPCa, detecting 97% (95% CI 80–116%; P = 0.023) of cases detected by mpMRI guided biopsies.

Similarly, the OPTIMUM trial was the first prospective RCT to demonstrate that micro‐US guided biopsy is non‐inferior to MRI/conventional US software‐fusion biopsy for the detection of csPCa. Micro‐US guided biopsy detected GG ≥ 2 cancers in 46% of patients compared to 43% with MRI/US fusion biopsy (difference, 3.52% [95% CI, −3.95% to 10.92%]; noninferiority P < 0.001).^46^ The use of micro‐US for PCa screening is also currently being investigated in a multicentre RCT (MUSIC‐Screen) trial comparing micro‐US and mpMRI.^47^

Whilst micro‐US has demonstrated potential advantages over MRI, there may be some limitations, including reduced detection ability for lesions in the transition zone and anterior prostate, especially in high‐volume prostates, due to the limited tissue penetration depth of 5 cm.^48^ This was demonstrated in a study where micro‐US findings were corroborated with radical prostatectomy findings. Whilst micro‐US overall showed a high sensitivity (20/23 [87%]) in detecting index lesions, the 3 missed lesions were all in the anterior transitional zone.^49^ Efforts have been made to overcome this limitation, including the development of novel protocols to detect PCa in the anterior zone.^50^

### 2.2 Molecular biomarkers

#### 2.2.1 Polygenic risk scores

PCa has a strong genetic component, and genome‐wide association studies (GWAS) have identified numerous common prostate cancer susceptibility loci.^51^ Polygenic risk scores (PRS) are individualised risk scores calculated as an aggregate risk from these DNA variants, which can vary from a dozen to thousands in number, to estimate the likelihood of developing the disease.^52^ PRS have been investigated in the context of risk‐stratified PCa screening for those individuals at the highest risk.

One UK study (N = 17 012, with 9494 PCa cases and 7608 controls) evaluated using a PRS consisting of 66 PCa susceptibility variants and found that the highest risk PRS quartile comprised 48% of PCa cases.^53^ With a PSA sensitivity of 80% and a mean sojourn time of 9 years, only 19% of screen‐detected cancers in the highest‐risk quintile were modelled to be overdiagnosed. This suggests that PRS could help address the issue of overdiagnosis and the subsequent overtreatment associated with PSA testing alone.

BARCODE1 is the first prospective study using PRS for PCa detection in primary care.^54^ A saliva‐derived PRS was calculated for participants, consisting of alleles for 130 risk loci. Of the 6142 men tested, 745 (12.1%) were invited for screening with MRI due to a PRS score >90th centile. Of the 468 men who underwent biopsy, 187 (40%) were diagnosed with PCa. Crucially, 103/187 (55%) of the PCa cases had a Gleason score >7, compared to 35.5% in the ERSPC study (p < 0.001). Furthermore, among those with a positive PRS score diagnosed with PCa, only 36.4% had an elevated PSA level (63.6% had a PSA level <3.0 μg/l).

The PROFILE study is examining targeted PCa screening for men at high risk, including men with a positive family history, men of Black ethnicity and those with high‐risk genetic variants such as BRCA1/2 mutations and/or a high PRS, defined as being in the top 10 percentile. Men will be given the choice of either undergoing an MRI and biopsy regardless of PSA levels or only if PSA exceeds a threshold of 1.0 ng/ml for those under 50 years old or 2.0 ng/ml for those aged 50 years or older. Early results from a feasibility study show that of 100 men with a family history of PCa who underwent biopsy, 25 (25%) were diagnosed with PCa, of which 12 (48%) had csPCa.^55^

#### 2.2.2 Blood‐based molecular biomarkers

##### Stockholm3

The Stockholm3 test uses a combination of clinical data (age, family history, previous prostate biopsy and prostate volume), 5 plasma protein markers (human glandular kallikrein 2, microseminoprotein beta, microphage inhibitory cytokine‐1, total PSA and free PSA) and a PRS based on genetic markers (single nucleotide polymorphisms) to assess for the risk of csPCa.^56^

Stockholm 3 has also been examined for PCa screening. A large prospective trial (N = 58 818) in Sweden compared the use of Stockholm3 versus PSA‐based population screening in men between the ages of 50–69 years.^57^ They demonstrated that a Stockholm3 cut‐off of 10% was greatly superior at detecting csPCa, as defined by Gleason score ≥7, with an AUC of 0·74 (95% CI 0·72–0·75) vs 0·56 (95% CI 0·55–0·60) (p < 0.001) for Stockholm3 and PSA alone, respectively. When using the same sensitivity as PSA cut‐off ≥3 ng/ml for high‐risk cancer, using Stockholm3 would have led to a reduction in the number of biopsies by 32% versus PSA alone.

Using a combination of PSA with Stockholm3 has been used to risk stratify patients pre‐MRI in a large (N = 12 750) prospective randomised trial.^58^ Compared to a PSA score ≥3, using a Stockholm3 cutoff score of 0.15 detected the same number of csPCa whilst reducing the number of MRI scans by 36% (545 vs 846; 0·64 [0·55–0·82]) and biopsies performed by 8% (311 vs 338; 0·92 (0·86–1·03). The formerly proposed cut‐off of ≥0.11 led to an 18% increased detection of csPCa but increased the number of performed MRIs and biopsies compared to PSA.

Whilst the Stockholm3 has mainly been tested in Scandinavia and more recently, in Denmark^59^ and a central European cohort (Switzerland and Germany),^60^ the test has also been validated in an ethnically diverse North American patient cohort (N = 2129), including 24% Black, 46% White, 14% Latino and 16% Asian men.^61^ Stockholm3 performance was consistent across ethnicities, with a greater overall AUC of 0.82 compared to PSA (AUC 0.66). Using a cut‐off score of ≥15 would have reduced biopsies of benign and clinically insignificant cancer by 45%. This is particularly important given the higher rates of incidence of PCa and cancer‐specific mortality in Black men in the UK and US,^62^, ^63^ necessitating the development of fair, inclusive and equitable PCa detection tests that can meet the needs of an ethnically diverse patient cohort.

##### 4Kscore® test

The 4Kscore is calculated using a combination of serum or plasma levels of four prostate‐specific kallikreins (Total PSA, Free PSA, Intact PSA and human kallikrein‐related peptidase 2) and clinical data (age, previous biopsy and DRE).^64^ It is approved by the U.S. Food and Drug Administration (FDA) for patients with an abnormal PSA or DRE to guide if a biopsy is warranted.^65^ The 4Kscore produces a score as a percentage which estimates the risk of aggressive disease (Gleason score ≥7).

In a large scale multicentre prospective validation trial (N = 1012) across 26 American centres, the 4 K score was compared to earlier risk models such as modified PCPTRC 2.0 (Prostate Cancer Prevention Trial Risk Calculator 2.0) for the detection of Gleason ≥7 tumours.^66^ The 4Kscore had a higher AUC compared to PCPTRC 2.0 with an AUC of 0.82 versus 0.74, p < 0.0001. Using a cutoff score of ≥6% would have avoided 307 (30%) of biopsies at the expense of delaying diagnosis for 13 of 232 Gleason ≥7 tumours. Furthermore, there were no major differences between the effectiveness of the score across ethnicities noted.

More recently, a large retrospective multi‐institutional study (N = 1111) compared using different strategies with 4Kscore and mpMRI prior to biopsy.^67^ Using the 4Kscore alone with a cut‐off score of ≥8 for biopsy would have avoided 260 (23.4%) biopsies at the expense of missing 13 (3.7%) of GG2 + tumours. Using a combination of 4Kscore (≥7.5 cutoff) and mpMRI led to a reduction in the number of biopsies avoided to 179 (16.1%) with only 4 (2.3%) GG2 + cancers missed.

The 4kscore was also evaluated as a reflex test for men with elevated PSA in the GÖTEBORG‐2 trial.^68^ In 571 men with PSA ≥ 3.0, the 4kscore (cutoff ≥7.5%) had an AUC of 0.84 (95% confidence interval 0.79–0.89) for intermediate to high risk PCa. Extrapolating this to per 1000 men tested, using a reflex 4k score would have led to 408(41%) fewer MRIs and 95(28%) fewer biopsies, at the expense of missing 4(4%) of intermediate grade cancers.

##### Prostate health index (PHI)

PHI is an FDA‐approved serum test designed to be used as an adjunct to PSA testing for men with elevated PSA levels (between 4 and 10 ng/ml) and a negative DRE.^69^ The PHI test consists of a combination of three kallikrein immunoassays (total PSA, free PSA and [−2]proPSA) to generate a score that predicts likelihood of PCa on biopsy.

In 2011 there was a multi‐centre trial (N = 892) that found that for men with elevated PSA (between 2 and 10 ng/ml), the PHI score had a greater ability to detect PCa with an AUC of 0.703 compared to an AUC of 0.525 for PSA.^70^ Furthermore, higher PHI scores were correlated with the risk of csPCa (e.g. score of ≥ 55 was 4.7‐fold higher risk than a score of <25).

The PHI score has also been evaluated in different ethnic groups, including African American men^71^ and Asian men.^72^, ^73^

#### 2.2.3 Urine‐based molecular biomarkers

##### ExoDx prostate (Intelliscore) test

The ExoDx is a urine test that does not require DRE; it measures exosomal RNA expression of 3 genes – *PCA3*, *ERG* and *SPDEF*.^74^ It is used in men over 50 years old with PSA levels 2–10 and who are being considered for initial/repeat biopsy. In an initial validation multi‐centre trial, ExoDx alone had a superior detection rate for high‐grade PCa than PSA, with an AUC of 0.74 (95% CI, 0.68–0.8) compared to an AUC of 0.55 (95% CI, 0.49–0.60) for PSA alone.^74^ For high‐grade disease (Gleason ≥7), using a cutoff score of 15.6 would have avoided 27% of prostate biopsies at the expense of missing only 8% of high‐grade tumours.

##### MyProstateScore

MyProstateScore (MPS) is a urine‐based test that is taken post DRE and it combines urinary prostate cancer antigen 3 (PCA3) and the *TMPRSS2:ERG* gene fusion (T2:ERG) with serum PSA.^75^ It has been validated for risk stratification for improving detection of GG ≥ 2 prior to biopsy.

In a large multicentre trial, with a retrospective training cohort (N = 677) and a prospective validation cohort (N = 1225), MPS had a greater detection rate for PCa overall and specifically for Gleason score >6 (AUC = 0.772) compared to PSA (AUC = 0.651), PSA plus T2:ERG (AUC = 0.747) and PSA plus PCA3 (AUC = 0.772).^76^

##### SelectMDx

SelectMDx is a post‐DRE urinary test that uses a combination of clinical data (age, DRE and PSA density) and mRNA expression of *DLX1* (Distal‐Less Homeobox 1) and *HOXC6* (Homeobox protein Hox‐C6) to estimate the risk of having Gleason ≥7 cancer. It is intended for risk stratification in men with elevated PSA prior to biopsy.^77^

The use of Select MDx alongside mpMRI has been studied.^78^, ^79^ In one multi‐centre prospective trial (N = 599) of men undergoing initial prostate biopsy, using SelectMDx only to risk stratify patients for biopsy would have avoided 227/599 (38%) of biopsies at the expense of missing 18/183 (13%) of csPCa.^80^ However, MRI‐only risk stratification was a superior strategy and would have avoided 295/599 (49%) of biopsies whilst missing only 9/183 (4.9%) csPCa.

### 2.3 Future directions

Efforts to enhance PCa testing access include innovations like paper‐based biomarker tests, enabling convenient point‐of‐care or at‐home screening with minimal blood droplets. These assays are less invasive than venous sampling, require smaller blood volumes and allow self‐testing, offering cost and time savings for patients.

Paper‐based biomarker analytics have been explored for PCa detection, with blood assays proving viable for distinguishing normal from elevated PSA levels.^81^, ^82^ However, these devices are not widely used due to poor correlation with venous PSA tests. Greater promise lies in capillary blood self‐sampling, as shown by an Amsterdam group using Topper technology and paediatric sample tubes for accurate PSA testing.^83^ Despite this, self‐sampling and paper‐based PSA tests share the same limitations as venous PSA testing. They may be more suited to resource‐poor regions with limited health infrastructure. Trials like PRAISE‐U will further evaluate self‐sampling in screening implementation.^84^

Multi cancer early detection (MCED) tests, which identify multiple cancers from a single blood sample.^85^ They offer potential for reducing cancer mortality, particularly where widespread screening is unavailable.^86^ One approach involves detecting circulating tumour DNA (ctDNA), as used in the Galleri and Cancerguard™ tests. The Galleri test analyses methylation patterns on cell‐free nucleic acids (cfNAs)^87^ and, in a North American study (N = 15 254), detected over 50 cancer types, including PCa with 99.5% specificity and 51.5% sensitivity across all cancer types.^88^ For PCa, results were less promising, with only 11.2% sensitivity (47/420) the second lowest among cancers. However, the test showed a bias towards detecting more csPCa, identifying 0% of GG1 tumours but 31.9% of GG4 and GG5 tumours.^89^ Given its low sensitivity, MCED tests may not be suitable for PCa screening, at least with methylation‐based approaches. The UK is currently evaluating the Galleri test in an RCT with over 140 000 participants,^90^ with results expected in 2026.^91^

A promising advancement in cancer detection is the use of circulating tumour cells (CTCs), which are tumour cells that detach from the primary site and enter the bloodstream.^92^ These cells are believed to contribute to the spread of cancer to distant organs and may be indicative of aggressive disease.^93^ CTC tests are already in clinical use, such as the CellSearch® system, which is FDA approved for monitoring metastatic PCa.^94^ Research suggests that CTC testing may have a role in the early detection of PCa. In one study (N = 600), men with a normal PSA but a positive CTC test, subsequent PSMA‐PET scans showed increased uptake in the prostate, indicating early PCa in 50% of those men.^95^ Another test (ISET®‐CTC test) has been examined as an adjunct to PSA testing.^96^ One study showed that of 27 men screened for PCa, 25 were CTC‐positive, with 20 of those men also having elevated PSA levels. Subsequent PSMA‐PET scans confirmed PCa in all 20 of those men. The combination of ISET®‐CTC and PSA testing demonstrated a positive predictive value (PPV) of 99% and a negative predictive value (NPV) of 97% for PCa.

Another innovation that holds promise for changing PCa testing is the introduction of artificial intelligence (AI) into the diagnostic pathway.

A small number of AI tools have been developed for the detection of PCa. One study compared the performance of a deep learning system (U‐Net) to clinical PI‐RADS assessment for detecting csPCa, finding that U‐Net achieved similar diagnostic performance and demonstrated potential to support clinical interpretation.^97^ Notably, two such tools, Quantib Prostate^98^ and ProstatID^99^ have already received FDA approval. Additionally, the Paige Prostate system, which assists with pathology interpretation, has received FDA approval, and the use of this system has been shown to lead to a 70% reduction in detection errors.^100^

## 3 CONCLUSIONS

PSA testing remains the primary tool for PCa detection, but its limited specificity and risk of overdiagnosis have led to the development of novel biomarkers and imaging techniques. MRI is now widely integrated into the diagnostic pathway, with mpMRI reducing overdetection of clinically insignificant PCa by nearly 50% in screening cohorts, as shown in the GÖTEBORG‐2 trial.^28^

However, mpMRI‐based screening has limitations, including cost, availability and unsuitability for some patients. Newer imaging modalities like bpMRI and mpUS show promise in addressing these issues while maintaining similar csPCa detection rates to mpMRI.^41^, ^101^ While MRI‐based screening trials are encouraging, evidence remains limited and this approach is resource‐intensive. Risk stratification using biomarkers or PRS before imaging may help, such as avoiding MRI in men with PSA < 1, given the low risk of csPCa and mortality.^102^ Additionally, biopsy methods will significantly impact diagnostic accuracy, underscoring the need for standardised approaches across screening modalities.

Looking ahead, emerging technologies such as PRS, MCEDs and CTC‐based tests show promise for early PCa detection. A combination of biomarkers, imaging, genetics, or AI may enable widespread, cost‐effective screening. However, the most effective approach remains unclear, requiring large‐scale trials in ethnically diverse cohorts to determine the optimal strategy. In the UK, PCa screening has gained renewed interest, with the UK NSC commissioning an analysis of six screening proposals, including risk‐stratified screening for high‐risk groups.^35^ Additionally, the UK National Screening Committee is involved in designing TRANSFORM, a national PCa screening trial for up to 300,000 men, set to begin recruitment in late 2024. The trial will assess various approaches, including different PSA thresholds, standalone MRI, MRI with PSA testing and polygenic risk scores (PRS), with the aim of identifying the most effective PCa screening method.^103^

## AUTHOR CONTRIBUTIONS

**Muhammad Haider:** Conceptualisation; writing—original draft; critical revision; final approval of the published version. **Jeffrey J. Leow:** Conceptualisation; writing—original draft; critical revision; final approval of the published version. **Tobias Nordström:** Conceptualisation; writing—original draft; critical revision; final approval of the published version. **Ashkan Mortezavi:** Conceptualisation; writing—original draft; critical revision; final approval of the published version. **Peter Albers:** Conceptualisation; writing—original draft; critical revision; final approval of the published version. **Rakesh Heer:** Conceptualisation; writing—original draft; critical revision; final approval of the published version. **Prabhakar Rajan:** Conceptualisation; writing—original draft; critical revision; final approval of the published version.

## CONFLICT OF INTEREST STATEMENT

Karolinska Institutet collaborates with A3P diagnostics in developing the technology for the Stockholm3 test. Tobias Nordström has stock ownership in A3P diagnostics. Rakesh Heer is a principal investigator on the TRANSFORM study.
